# Evaluation of Antioxidant and Antibacterial Activities of Aqueous, Methanolic and Alkaloid Extracts from *Mitragyna Speciosa* (Rubiaceae Family) Leaves 

**DOI:** 10.3390/molecules14103964

**Published:** 2009-10-09

**Authors:** Suhanya Parthasarathy, Juzaili Bin Azizi, Surash Ramanathan, Sabariah Ismail, Sreenivasan Sasidharan, Mohd Ikram Mohd. Said, Sharif Mahsufi Mansor

**Affiliations:** 1 Centre for Drug Research, Universiti Sains Malaysia, 11800 Minden, Penang, Malaysia; 2 Institute for Research in Molecular Medicine, Universiti Sains Malaysia, 11800 USM, Pulau Penang, Malaysia; 3 School of Chemical Sciences and Food Technology Universiti Kebangsaan Malaysia 43600 Bangi, Selangor, Malaysia

**Keywords:** *Mitragyna speciosa*, antioxidant, antibacterial

## Abstract

Studies on the antioxidant and antimicrobial activities of *Mitragyna speciosa* leaf extracts are lacking. In this study the antioxidant properties of water, methanolic and alkaloid *M. speciosa* leaf extracts were evaluated using the DPPH (2,2-diphenyl-1-picrylhydrazyl) radical scavenging method. The amount of total phenolics and flavanoid contents were also estimated. The DPPH IC_50_ values of the aqueous, alkaloid and methanolic extracts were 213.4, 104.81 and 37.08 μg/mL, respectively. The total phenolic content of the aqueous, alkaloid and methanolic extracts were 66.0 mg, 88.4, 105.6 mg GAE/g, respectively, while the total flavanoid were 28.2, 20.0 and 91.1 mg CAE/g respectively. The antioxidant activities were correlated with the total phenolic content. This result suggests that the relatively high antioxidant activity of the methanolic extract compared to aqueous and alkaloid extract could be possibly be due to its high phenolic content. The aqueous, alkaloid and methanolic extracts were screened for antimicrobial activity. The extracts showed antimicrobial activity against *Salmonella typhi* and *Bacillus subtilis.* The minimum inhibitory concentrations (MICs) of extracts determined by the broth dilution method ranged from 3.12 to 6.25 mg/mL. The alkaloid extract was found to be most effective against all of the tested organisms.

## 1. Introduction

Natural products, such as plant extract, either as pure compounds or as standardized extracts, provide unlimited opportunities for new drug discoveries because of the unmatched chemical diversity the can provide [1]. According to the World Health Organization (WHO), more than 80% of the world's population relies on traditional medicine for their primary healthcare needs. This has captured the interest of many researchers to explore local medicinal plants for valuable medicinal traits. Several studies indicate that medicinal plants contain compounds like peptides, unsaturated long chain fatty acids, aldehydes, alkaloids, essential oils, phenols and water or ethanol soluble compounds. These compounds are significant in therapeutic application against human and animal pathogens, including bacteria, fungi and viruses [2,3]. In recent years, numerous drug resistances in human pathogenic microorganisms have developed due to the indiscriminate use of commercial antimicrobial drugs commonly used in the treatment of infectious diseases. On the other hand, free radicals are known to be the major cause of various chronic and degenerative diseases. Oxidation is a natural process in organisms for the production of energy to fuel biological cycles. Conversely, the uninhibited production of oxygen-derived free radicals is involved in the onset of many diseases such as arthritis, atherosclerosis, rheumatoid and cancer as well as in many degenerative diseases related with aging [4]. This situation has prompted the continuous search for various plant sources with these medicinal values. *Mitragyna speciosa* is a tropical plant indigenous to the Northern Malay Peninsula and Thailand. The leaves of this plant are, known as “Biak” and “Ketum” in Malaysia, and as “Kratom” in Thailand. Mitragynine has been suggested as a useful constituent in the treatment of opiate addiction as a replacement therapy [5]. The leaves has been also reported for its, antitussive, anaesthetic ,antinociceptive, stimulant, analgesic and narcotic-like actions properties [5,6,7]. However, to date no research on antimicrobial and antioxidant properties of the *M. speciosa* have been documented. In this study we report the antimicrobial and antioxidant activities of aqueous, methanolic and alkaloids extracts of *M. speciosa* leaf.

## 2. Results and Discussion

Existing literature mainly reports the antitussive, anaesthetic, antinociceptive, stimulant, analgesic and narcotic-like action pharmacological activities of *M. speciosa* leaves. These pharmacological activities are often attributed to the alkaloids found in the leaf [6]. Mitragynine is the dominant alkaloid and is exclusive to *M. speciosa*. To date, studies on the antioxidant and antimicrobial properties are limited and not thoroughly documented. In this study the methanolic, alkaloid and aqueous extracts of the plant leaves were evaluated for their antioxidant and antimicrobial properties.

### 2.1. Total phenolic and flavanoid content

Table 1 shows the total phenolic and flavanoid contents in the *Mitragyna speciosa* leaf extracts. The highest (P < 0.05) amount of phenolic content was found in the MeOH extract (105.58 ± 15.43 mg/g) followed by the alkaloid extract (88.37 ± 0.70 mg/g) and aqueous (66.00 ± 1.23 mg/g) extract. The flavanoid contents were the most (P < 0.05) in the MeOH extract (91.12 ± 17.27 mg/g) and the least was found in aqueous (28.19 ± 2.28 mg/g) and alkaloid extracts (20.03 ± 3.03 mg/g).

### 2.2. Antioxidant assays

The antioxidant activity of the extracts was determined using a DPPH scavenging assay. The DPPH assay is often used to evaluate the ability of antioxidants to scavenge free radicals which are known to be a major factor in biological damages caused by oxidative stress. This assay is known to give reliable information concerning the antioxidant ability of the tested compounds [8]. The principle of the assay is based on the color change of the DPPH solution from purple to yellow as the radical is quenched by the antioxidant [9]. The methanol extract of the leaf exhibited a significant dose dependent inhibition of DPPH activity (P < 0.05), with a 50% inhibition (IC_50_) at a concentration of 37.08 ± 3.54 µg/mL (Table 1).

**Table 1:** The total phenolic, flavanoid content and DPPH IC_50_ of various *Mitragyna speciosa* leaf extract.

**Extract**	Total phenolic content mg/g	Total flavanoid content mg/g	IC _50_µg/mL
**MeOH extract**	**105.58 ± 15.43**	**91.12 ± 17.27**	**37.08 ± 3.54**
**Aqueous extract**	**66.00 ± 1.23**	**28.19 ± 2.28**	**213.45 ± 31.31**
**Alkaloid extract**	**88.37 ± 0.70**	**20.03 ± 3.03**	**104.81 ± 5.77**
**BHT**	**−**	**−**	**4.50 ± 0.23**

P < 0.05.

The corresponding IC_50_ for alkaloid and water extracts were 104.81 ± 5.77 µg/mL and 213.45 ± 31.31 µg/mL respectively. The methanolic extract IC_50_ value was low compared to other extracts but several fold higher than that of the reference compound butylated hydroxytoluene (BHT), which had an IC_50_ value of 4.5 µg/mL. The high content of phenolic compounds in the methanolic extract might explain its higher antioxidant capacity compared to that of other extracts. There is evidence of a good correlation between phenolic contents of the different extracts (R^2^ = 0.9833) and their IC_50_ DPPH values. These results were consistent with the findings of many research groups who reported such correlation between total phenolic content and IC_50_ antioxidant activity [10,11,12,13]. The alkaloid extract DPPH IC_50_ was two-fold higher than the aqueous extract while their total phenolic and flavanoid contents were not much different. The Folin–Ciocalteu assay used herein provides a crude estimation of the total phenolic compounds present in an extract. Since it is not specific to polyphenols any interfering compounds may react with the reagent and thus leads to noticeable elevated phenolic concentrations [14]. In addition, phenolic compounds respond differently in this assay when the number of phenolic groups varies [15]. In this study the DPPH assay was utilized to determine the antioxidant activity. The DPPH assay involves a reduction mechanism, therefore it is possible that the aqueous leaf extract probably had lesser DPPH reductones compared to that of the alkaloids extract but reacts with the Ciocalteu reagent in the assay. These might explain the marked differences in DPPH IC_50_ between the aqueous and alkaloid extracts while the phenolic content was comparable. Apart from this, variation in the antioxidant mechanism of active compounds in the extracts could also lead to these differences [16].

### 2.3. Antimicrobial activity

Preliminary antimicrobial screening assay of the *M. speciosa leaf* extracts is shown in both methanolic and alkaloid extracts gave relatively wide inhibition zones against the test strains compared with the positive control. The relatively wider spectrum of activity of the methanol/alkaloid extracts over the positive control is significant from the disk diffusion assay. The inhibition zones of the *M. speciosa* leaf methanolic (29–30 mm) and alkaloid extracts (30–33 mm) were greater than (P < 0.05) that of the positive control (23–24 mm). No inhibition zones were observed for the aqueous extracts for all bacterial strains tested. The aqueous extract and negative control methanol were devoid of any antimicrobial activity. After the antimicrobial screening assay, the extracts that showed positive results were selected for further studies for the determination of MIC. The methanolic and alkaloid extracts MICs for the tested bacterial strains were 6.25 mg/mL and 3.25 mg/mL respectively. In this study both methanolic and alkaloid extracts demonstrated antibacterial activity which may explain anonymous claim on the topical use of *M. speciosa* leaves for infected wound.

**Table 2:** Antimicrobial activity (zone of inhibition and MIC*^a^*) of *Mitragynaspeciosa* Leaf extract compared with commercial antibiotic 30 *μ*g/mLchloramphenicol.

Microorganism	Zone of inhibition (mm)^a^					MIC (mg/mL)^b ^of the extract		
	MeOH extract	Aqueous extract	Alkaloid extract	Chloramphenicol	MeOH extract		Aqueous extract	Alkaloid extract
*Salmonella typhi*	29 ± 2.3	-	30 ± 2.1	23	6.25		-	3.12
*Bacillus subtilis*	30 ± 2.1	-	33 ± 2.5	24	6.25		-	3.12
*Escherichia coli*	-	-	-	22	-		-	-
*Pseudomonas aeruginosa*	-	-	-	23	-		-	-

^a^ The values (average of triplicate) are diameter of zone of inhibition at 100 mg/mL; ^b^ Broth dilution method, mean value n = 3, P < 0.05.

**Figure 1:**
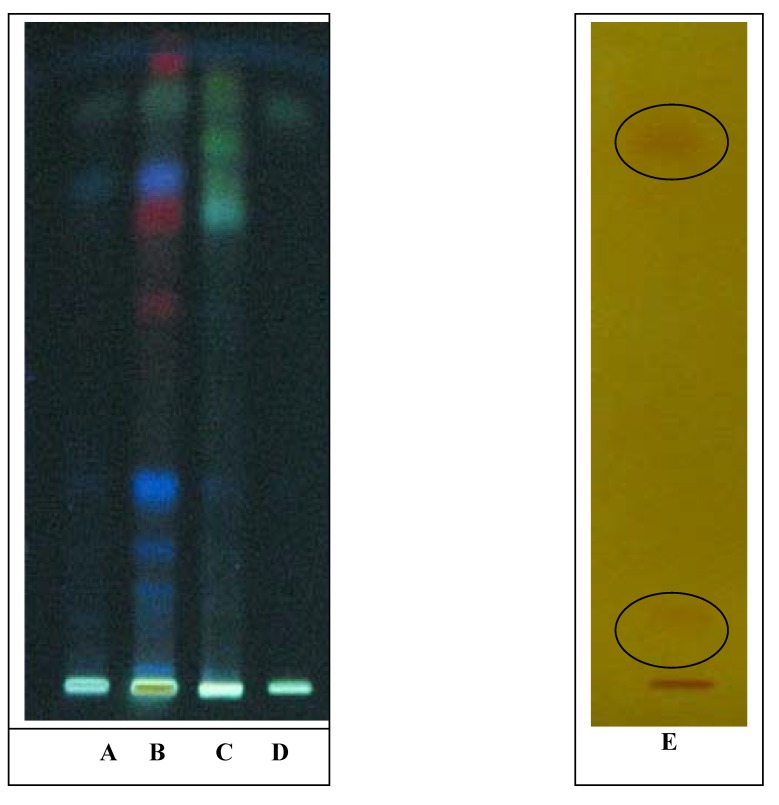
TLC Chromatogram of: (A) aqueous Extract; (B) alkaloid extract; (C) methanolic extract; (D) mitragynine standard, visualized at 365 nm; (E) alkaloid extract visualize by Dragendorff.

High performance thin layer chromatography (HPTLC) method was used to detect the presence of bioactive compounds in the alkaloid, methanolic and water extracts as this method provides a rapid detection and localization of the active compounds in a plant extract. The extracts were dissolved in methanol and applied to silica gel plates. Mitragynine was developed using the solvent system (ethyl acetate: hexane (4:1) and the separated zones were visualized under UV light (365 nm). For all extracts screened a green spot with Rf value of 0.92 was identified as mitragynine in comparison with mitragynine standard that had the same Rf value for standard solution spotted on the TLC plate (Figure 1). This indicates the presence of mitragynine in all extracts but the intensity of the spots varies. The mitragynine spot was more visible in alkaloid and methanolic extract than that of the aqueous extract. Alkaloid extract had several visible colour spots observed on the TLC plate (Figure 1) Two brown colored spots were visualized after the TLC plate was sprayed with Dragendorff-H_2_SO_4_ solution thus indicating the presence of alkaloids.

## 3. Experimental

### 3.1. Plant material

The fresh plant leaves of *M. speciosa* were collected from the state of Perak Malaysia. An initial quality evaluation of the plant material was carried out to validate its authenticity and also to ensure quality, using techniques adopted from the WHO Guidelines on Herbal Quality Control [18]. The authentication work was done by a botanist from the School of Biological Sciences, Universiti Sains Malaysia where the plant material was deposited. The leaves were washed with water to remove dirt prior to the drying process. The plant material were cut, air-dried and grounded into powder.

### 3.2. Chemicals

2,2-Diphenyl-1-picryl-hydrazyl (DPPH), Folin-Ciocalteu reagent, aluminium chloride (AlCl_3_),gallic acid, ascorbic acid and catechin were purchased from Sigma-Aldrich USA. Butylated hydroxytoluene (BHT) was purchased from Fluka. Sodium sulphate and sodium hydroxide were purchased from Bendosen and System, respectively. Sodium phosphate and sodium carbonate was obtained from Acros Organics. HPLC grade methanols, glass silica gel sheets, were purchased from Merck. Ethyl acetate, hexane acetic acid, chloroform, petroleum ether and were purchased from Fisher Scientific.

### 3.3. Plant extraction procedure

#### 3.3.1. Hot water extraction

Five kilograms of dried MG leaves was boiled in eight litres of water for two hours, and then drained and reboiled for the second time at 100 ^ο^C for 2 hours. The aqueous extract was blow dried to give 375 g of the extract.

#### 3.3.2. Methanolic and alkaloid extract

Dry, powdered leaves were soaked in methanol for several days at room temperature. The mixture was filtered and methanol was removed by rotary evaporator to give the crude methanolic extract. Procedure of extraction and evaporation was repeated three times. As for preparation of crude alkaloid mixture, the methanol extract was mixed with acetic acid-water (9:1), 1 part methanol extract to 35 parts acetic acid. Alternatively acid extraction was done by triturating the methanol extract with 5% aq. HCl. The suspension was then filtered and the filtrate was washed with petroleum ether. The acidic layer was basified with sodium carbonate to pH 9 and extracted with chloroform several times. The combined chloroform extract was dried over sodium sulphate and evaporated to give the crude alkaloid mixture.

### 3.4. Standardize extracts

All *M. specioasa* leaf extracts were standardized in reference to mitragynine content using validated HPLC assay method. Standardized water, methanolic and alkaloid leaf extracts were used for antimicrobial and antioxidant studies.

### 3.5. Test microorganisms and growth media

The following Gram-positive and Gram-negative bacteria, was used for antimicrobial activities studies: bacteria included *Salmonella typhi, Bacillus subtilis, Escherichia coli, Pseudomonas aeruginosa*. The bacterial strains were grown in Mueller–Hinton agar (MHA) plates at 37 °C. The stock culture was maintained at 4 °C.

### 3.6. Antioxidant assay

#### 3.6.1. Total phenolic assay

Total Phenolics (TPC) in the extracts was determined using the Folin-Ciocalteu reagent method [19]. This method depends on the reduction of FCR by phenols to a mixture of blue oxides which have a maximal absorption in the region of 765 nm using spectrophotometer (UNIDEC-50), Jasco Corporation, Tokyo, Japan*.* Stock solution of MG leaf extracts were prepared to the concentration of 1mg/mL (5 mg of powdered extract dissolved in 5 mL of ethanol). To 1.0 mL of each extract, 5 mL of Folin-Ciocalteu Reagent were added. The mixture solution was vortexed and incubated in the dark for 3 minutes, respectively. After that 5 mL of sodium carbonate (75g/L) solution was added to the mixture and mixed thoroughly. The mixture was incubated in the dark for 1 hour. The absorbance was read at 765 nm. Blank consisted of 5 mL Folin-Ciocalteu reagent, 1 mL ethanol and 4 mL sodium carbonate solution. The total phenolic compounds concentration in the extract was expressed as milligrams of gallic acid equivalent per gram of dry weight (mg GAE/g) of extract. Gallic acid stock solution was prepared by diluting 10 mL of gallic acid powder into 10 mL of ethanol (1 mg/mL). Serial dilution was carried out; gallic acid solution was dissolved in ethanol. A linear dose- response regression curve was generated using absorbance reading of gallic acid at the wavelength of 765 nm. All determinations were carried out in triplicates. Results were expressed as milligrammes of gallic acid equivalent per gram of dry weight (mg GAE/g) of extracts. Total content of phenolic compounds in the plant extract was calculated using this formula; total phenolic content = GAE * V/m. Where GAE, is the Gallic Acid Equivalence (mg/mL) or concentration of gallic acid established from the calibration curve; V, is the volume of extract (mL) and m, is the weight of pure plant extract (g).

#### 3.6.2. Total flavonoid content

The method described by [20] was used as a guideline to carry out this determination. The flavonoid content was determined by aluminium chloride method using catechin standard solution. Extract or (+) catechin standard solution was prepared in ethanol (1 mg/mL). A calibration curve using various catechin concentrations (0.5, 0.3, 0.1, 0.08 and 0.02 mg/mL) was obtained. 0.25 mL of extract or (+) catechin standard solution was mixed with 1.25 mL of distilled water in test tubes, followed by addition of 75 µL of a 5% sodium nitrite solution. After six minutes, 150 µL of a 10% aluminium chloride solution was added and the mixture was allowed to stand for further five minutes after which 0.5 mL of 1M sodium hydroxide was added to the solution. The mixture was brought to 2.5 mL with distilled water and mixed well. Blank consist of all the reagents, except for the extract or catechin standard solution is substituted with 0.25 mL of ethanol. The absorbance was measured immediately at 510 nm using a spectrophotometer. Determination was done in three replicates. Results were expressed as milligrammes of catechin equivalent per gramme of dry weight (mg CE/g) of extracts. Total content of flavonoid compounds in the plant extract was calculated using this formula:

Total flavonoid content = CE * V/m

Where CE, is the catechin equivalence (mg/mL) or concentration of catechin solution established from the calibration curve; V, is the volume of extract (mL) and m, is the weight of the pure plant extract (g).

#### 3.6.3. 1,1-Diphenyl-2-picrylhydrazyl (DPPH) assay

The scavenging activity of *Mitragyna speciosa* leaf extracts on DPPH was determined using the method described by [21]. This method depends on the reduction of purple DPPH to a yellow colored diphenyl picrylhydrazine. The determination of the disappearance of free radicals was done using spectrophotometer [22].The remaining DPPH which showed maximum absorption at 518 nm was measured. Each plant extract sample’s stock solution (1.0 mg/mL) was diluted to final concentrations of (0.6 .0.5, 0.4, 0.3, 0.2, 0.1, 0.05 and 0.01) mg/mL, in ethanol. One mL of a 0.3 mM DPPH ethanol solution was added to 2.5 mL of sample solution of different concentrations. These are test solutions. One ml of ethanol was added to 2.5 mL of sample solution of different concentration. These are blank solutions. One mL DPPH solution plus 2.5 mL of ethanol was used as a negative control. The blank for this solution is ethanol. As DPPH is sensitive to light, it is exposed to the minimum possible light. These solutions were allowed to react at room temperature for 30 minutes.

The absorbance values were measured at 518 nm and converted into the percentage antioxidant activity using the following equation:

Scavenging capacity (%) = 100 – [(absorbance of sample – absorbance of blank) × 100/absorbance of control]

The tests were done in triplicate. The IC_50_ values were calculated by linear regression of plots, where the abscissa represents the concentration of the tested plant extracts and the ordinate the average percent of scavenging capacity. The concentration of sample required to scavenge 50% of DPPH (IC_50_) were determined.

### 3.7. Antimicrobial assay

#### 3.7.1. Disk diffusion assay

Antibacterial activities of the different extracts were investigated by the disk diffusion method [23,24]. The MHA plates, containing an inoculum size of 10^6^ colony-forming units (CFU)/mL of bacteria or 2 × 10^5^ CFU/mL yeast cells or molds spores on SDA and PDA plates, respectively, were spread on the solid plates with an L-shaped glass rod. Then disks (6.0-mm diameter) impregnated with 25 µL of each extract at concentrations of 100.0 mg/mL were placed on the inoculated plates. Similarly, each plate carried a blank disk by adding solvent alone in the center to serve as a control, and antibiotic disks (6.0-mm dia) of 30 μg/mL chloramphenicol was also used as positive controls. All the plates were incubated at 37 °C for 18 to 24 h. The zones of growth inhibition around the discs were measured after 18 to 24 h of incubation at 37 °C. The sensitivity of the microorganism species to the plant extracts was determined by measuring the sizes of inhibitory zones (including the diameter of disk) on the agar surface around the disks, and values <8 mm were considered as not active against microorganisms. All of the experiments were performed in triplicate. The results are reported as the average of three experiments.

#### 3.7.2. Determination of minimum inhibitory concentrations (MIC)

A 16 h culture was diluted with a sterile physiological saline solution [PS; 0.85% (w/v) sodium chloride] with reference to the 0.5 McFarland standards to achieve an inoculum size of approximately 10^6^ colony forming unit mL^-1^. A serial dilution was carried out to give final concentrations between 1.563-200.00 mg crude extract per mL. The tubes were inoculated with 20 μL of the bacterial suspension per mL nutrient broth, homogenized and incubated at 37 °C. The MIC value was determined as the lowest concentration of the crude extract in the broth medium that inhibited the visible growth of the test microorganism [25].

### 3.8. Statistical analysis

All the assays were carried out in triplicate. Experimental results were expressed as means ± standard deviation (SD) of three parallel measurements. The differences between the extracts were analyzed using one-way analysis of variance (ANOVA) with *α* = 0.05. This statistical analysis was carried out using the SPSS version 12 programmer.

## 4. Conclusion

In conclusion, the screening of antioxidant and antimicrobial activity performed on *M. speciosa* leaf extracts which was traditionally used as herbs shows that they are endowed with potentially exploitable free radical scavenging and antimicrobial activity. Hence, *M. speciosa* leaf could be used as an easy accessible source of natural antioxidants and antimicrobial agent. Further purification of the active compounds and *in vivo* evaluation of antioxidant and antimicrobial activity along with toxicity studies of the extracts from *M. speciosa* are therefore suggested for further studies.
